# The moderating role of hippocampus-dorsolateral prefrontal cortex resting-state functional connectivity in the relationship between emotional abuse and depression in adolescents

**DOI:** 10.1192/j.eurpsy.2025.447

**Published:** 2025-08-26

**Authors:** K. H. Lee, J. Shin, J. Lee, J. H. Yoo, J.-W. Kim

**Affiliations:** 1 Psychiatry, Seoul National University Hospital, Seoul; 2 Psychiatry, Eulji University School of Medicine, Uijeongbu; 3 Integrative Care Hub, Seoul National University Children’s Hospital; 4 Psychiatry, The Catholic University of Korea, Seoul ST. Mary’s Hospital, Seoul, Korea, Republic Of

## Abstract

**Introduction:**

Early life adversity (ELA) such as physical and emotional abuse has been recognized as an important risk factor for depression in adults. Past research has shown that ELA was associated with alteration in the hippocampus, a key region involved in stress sensitivity, emotional learning and memory. However, relatively little is known about the role of the hippocampus in the relationship between ELA and depression in adolescents.

**Objectives:**

This study aimed to investigate whether the hippocampal volume and hippocampus resting-state functional connectivity (RSFC) moderated the relationship between ELA and depressive symptom severity in adolescents with major depressive disorder (MDD).

**Methods:**

This study included 73 adolescents with MDD (age M (SD) = 15.0 (1.5) years, 51 girls). The participants completed the Early Trauma Inventory and Children’s Depression Rating Scale to assess ELA and depressive symptom severity, respectively. Resting–state functional and structural T1 images were collected using a Siemens 3T MR scanner and preprocessed using AFNI and FreeSurfer routines. The average BOLD time-series was extracted from our regions-of-interest (ROIs), the bilateral hippocampus and dorsolateral prefrontal cortex (DLPFC). An ROI-to-ROI RSFC analysis was conducted to calculate Pearson correlation coefficients between the hippocampus and DLPFC ROIs. The correlation coefficients were transformed to Fisher’s z. We performed correlation and moderation analyses to test our moderation model (Figure 1) after controlling for age and sex.

**Results:**

Emotional abuse, one form of ELA, was significantly correlated with depressive symptoms in adolescents with MDD (*r* = 0.25, *p* < .05). Bilateral hippocampus – left DLPFC RSFCs moderated the association between emotional abuse and depressive symptoms in adolescents with MDD (*ps* < .01). The association between emotional abuse and depressive symptoms was stronger when bilateral hippocampus – left DLPFC RSFCs were lower (left hippocampus – left DLPFC RSFC, -1D: *b* = 3.72, *SE* = 1.06, *p* < .001; right hippocampus – left DLPFC RSFC, -1D: *b* = 4.15, *SE* = 1.04, *p* < .001) than when they were greater (left hippocampus – left DLPFC RSFC, +1D: *b* = -0.09, *SE* = 1.05, *p* = .93; right hippocampus – left DLPFC RSFC, +1D: *b* = -0.10, *SE* = 0.98, *p* = .69) (Figure 2). Hippocampal volumes also moderated the relationship between emotional abuse and depressive symptoms, but the results did not remain significant after correcting for multiple comparisons.

**Image 1:**

**Figure:**
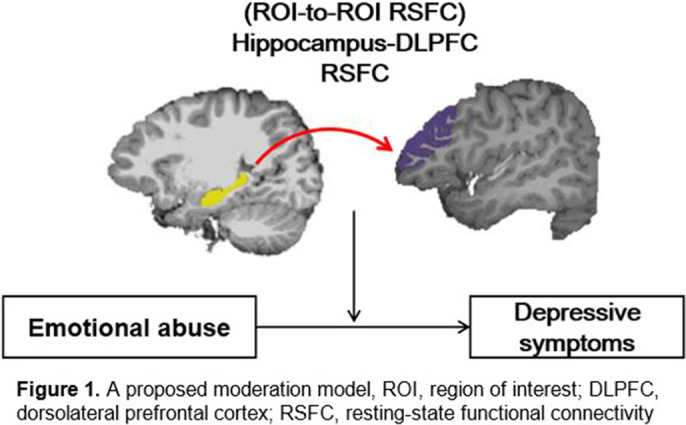

**Image 2:**

**Figure:**
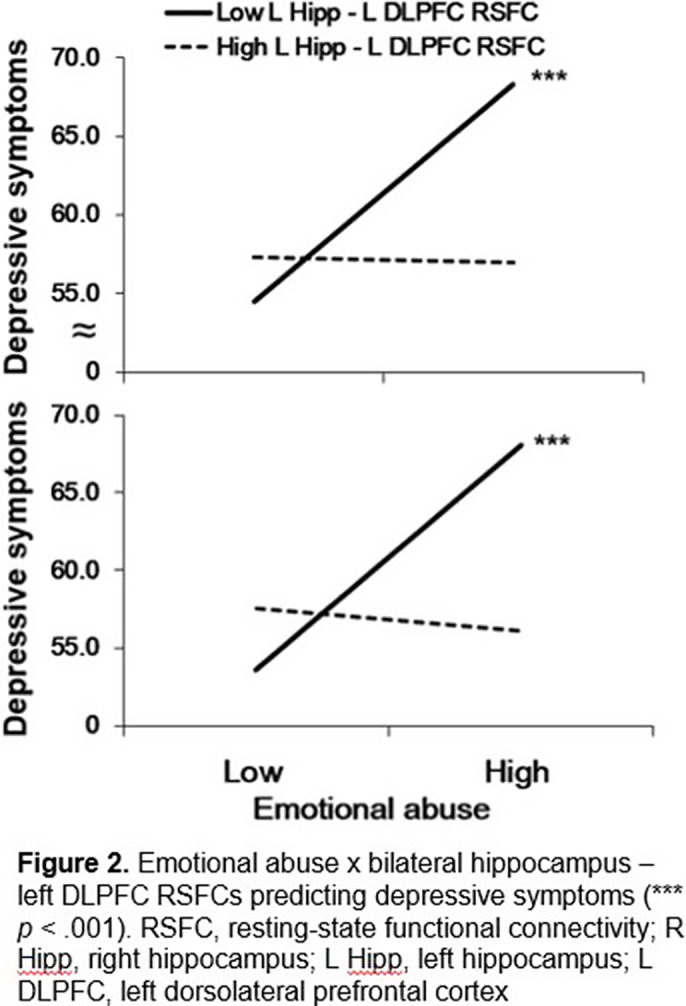

**Conclusions:**

Our findings suggest the important role of hippocampus RSFC with the DLPFC in the relationship between emotional abuse and depressive symptoms in adolescents with MDD.

**Disclosure of Interest:**

None Declared

